# Canadian national electrophysiology ablation registry report 2011–2016

**DOI:** 10.1186/s12913-021-06441-0

**Published:** 2021-05-06

**Authors:** Anna Kaoutskaia, Mohammed Shurrab, Guy Amit, Ratika Parkash, Derek Exner, Satish Toal, Laurence Sterns, Jean-Francois Sarrazin, Vijay Chauhan, Omar Sultan, Girish Nair, Marc Deyell, Laurent Macle, Steve Klassen, Benedict Glover, Eugene Crystal

**Affiliations:** 1 Sunnybrook Health Sciences Centre, University of Toronto, 890 Sheppard Avenue West, Ontario M4N 3M5 Toronto, Canada; 2 St. Matthew’s University School of Medicine, Grand Cayman, Cayman Islands; 3 Health Sciences North, Sudbury, Ontario Canada; 4 McMaster University, Hamilton, Ontario Canada; 5 Dalhousie University, Halifax, Nova Scotia Canada; 6 University of Calgary, Calgary, Alberta Canada; 7 Saint John Regional Hospital, Saint John, New Brunswick, Canada; 8 Royal Jubilee Hospital, Victoria, British Columbia Canada; 9 Université Laval, Quebec City, Quebec Canada; 10 Toronto General Hospital, Toronto, Ontario Canada; 11 Regina General Hospital, Regina, Saskatchewan Canada; 12 University of Ottawa Heart Institute, Ottawa, Ontario Canada; 13 University of British Columbia, Vancouver, British Columbia Canada; 14 Montreal Heart Institute, Montreal, Quebec Canada; 15 St. Boniface Hospital, Winnipeg, Manitoba Canada

**Keywords:** Cardiac catheter ablation, Survey, Registry, Invasive electrophysiology, Arrhythmia

## Abstract

**Background/purpose:**

: Interventional cardiac electrophysiology (EP) is a rapidly evolving field in Canada; a nationwide registry was established in 2011 to conduct a periodic review of resource allocation.

**Methods:**

The registry collects annual data on EP lab infrastructure, imaging, tools, human resources, procedural volumes, success rates, and wait times. Leading physicians from each EP lab were contacted electronically; participation was voluntary.

**Results:**

All Canadian EP centres were identified (*n* = 30); 50 and 45 % of active centres participated in the last 2 instalments of the registry. A mean of 508 ± 270 standard and complex catheter ablation procedures were reported annually for 2015–2016 by all responding centres. The most frequently performed ablation targets atrial fibrillation (PVI) arrhythmia accounting for 36 % of all procedures (mean = 164 ± 85). The number of full time physicians ranges between 1 and 7 per centre, (mean = 4). The mean wait time to see an electrophysiologist for an initial non-urgent consult is 23 weeks. The wait time between an EP consult and ablation date is 17.8 weeks for simple ablation, and 30.1 weeks for AF ablation. On average centres have 2 (range: 1–4) rooms equipped for ablations; each centre uses the EP lab an average of 7 shifts per week. While diagnostic studies and radiofrequency ablations are performed in all centres, point-by-point cryoablation is available in 85 % centres; 38 % of the respondents use circular ablation techniques.

**Conclusions:**

This initiative provides contemporary data on invasive electrophysiology lab practices. The EP registry provides activity benchmarks on national trends and practices.

**Supplementary Information:**

The online version contains supplementary material available at 10.1186/s12913-021-06441-0.

## Background

Characterization and reporting of resources in health care is vital for means allocation, designing new facilities, and policy making. Interventional cardiac electrophysiology (EP) is a rapidly evolving field. Ablation technologies to treat cardiac dysrhythmias became widely available and affordable. Over the past decades, the number of centres in Canada that perform catheter ablations of arrhythmias and other invasive EP procedures has significantly augmented. An increasing number of certified electrophysiology specialists are entering the field as indications for complex ablations are growing. We established the first Canadian nationwide cardiac ablation registry in 2011, to understand the variability in the conduct of EP procedures and resource allocation across the country. The purpose of this publication is to provide a 6-year update on national practices of invasive cardiac EP.

## Methods

The “EP Ablation Registry” survey was developed for this project by an independent steering committee in collaboration with the ablation committee of Canadian Heart Rhythm Society (CHRS**)** and with funding support from International Winter Arrhythmia School (IWAS). Since the registry’s inception in 2011, we continuously identified and added EP centres to our database through the CHRS, IWAS symposium and industry liaisons. There is an EP lab in most major university-affiliated hospitals; and 120 practicing interventional cardiac electrophysiologists, performing standard and complex ablations that serve Canada’s 37 million population [1]. Active directors and leading physicians in EP centres were contacted electronically; participation in the survey was voluntary.

The initial questionnaire was sent out electronically for retrospective completion to all identified laboratories. Design of the questionnaire was expanded upon after its first installment, which is described in detail elsewhere [1].

### Data analysis

Discrete numerical results are expressed as totals and means ± standard deviation. Categorical variables expressed as number and percentage. Ranges are reported where applicable. Statistical analyses were completed using a Microsoft Excel version 15.31 package.

## Results

### Response rate

In 2017, we identified 30 active adult EP labs in Canadian centres where cardiac ablations are performed; that number increased from 25 in the first survey installment. The response rate was 76 % in original survey sent in 2012, 50 % in the second installment in 2014, and 47 % in 2016. Eleven centres have been consistently reporting their data in all 3 surveys, and are used in this analysis when comparing same data categories.

The identified EP centres are located in 9 of 13 provinces and territories; Prince Edward Island and three territories are served by external EP labs, typically located in neighboring provinces.

### Laboratory Infrastructure and human resources

Table 1 provides a comparison of the infrastructure and resources trends 2011 to 2016 from all contributing centres.

**Table 1:** Facility Resource Information

	2015–2016	2013–2014	2011–2012
	N (%)	N (%)	N (%)
University Affiliated Hospitals	14 (100 %)	14 (100 %)	17 (89 %)
Lab Infrastructure			
Centers: Implants done in OR	4 (29 %)	6 (43 %)	8 (47 %)
Ablation rooms	1.9 (1–4)^a^	1.8 (1–3)^a^	1.6 (1–3)^a^
Days/Week Room used for Ablation^c^	7.0 (2–14)^a^	7.1 (2–14)^a^	5.3 (1–13)^a^
Implant Days/Week^c^	3.4 (0–5)^a^	3.2 (0–5)^a^	3.25 (1–6)^a^
EP Procedures Performed			
Diagnostic EP Procedures	14 (100 %)	14 (100 %)	19 (100 %)
RF	14 (100 %)	13 (93 %)	19 (100 %)
Contact Force	14 (100 %)	n/a	n/a
Cryoablation point-by-point	12 (86 %)	9 (64 %)	n/a
Cryoballoon	10 (71 %)	6 (43 %)	15 (83 %)
PVAC	3 (21 %)	4 (29 %)	n/a
NMARQ (circular RF)	2 (14 %)	0	n/a
Mapping Systems			
CARTO	12 (86 %)	13 (93 %)	14 (78 %)
Intracardiac echo	12 (86 %)	12 (86 %)	14 (78 %)
EnSite-NavX Velocity	10 (71 %)	13 (93 %)	15 (83 %)
EnSite-NavX Precision	8 (57 %)	n/a	n/a
Mediguide	2 (14 %)	2 (14 %)	n/a
Rhythmia™	1 (7 %)	0	n/a
LocaLisa	1 (7 %)	1 (7 %)	1 (6 %)
STXS	2 (14 %)	1 (7 %)	2 (11 %)
Imaging Systems^b^			
General Electric	1 (7 %)	1 (7 %)	n/a
Philips	8 (57 %)	8 (57 %)	n/a
Siemens	6 (43 %)	7 (50 %)	n/a
Toshiba	2 (14 %)	2 (14 %)	n/a
Portable GE	1 (7 %)	1 (7 %)	n/a

^a^ = range

^b^ = present in number of centres

^c^ = days per week room used for ablation x number of rooms

Data in the latest survey (n = 14) was from all university affiliated hospitals that perform EP studies and catheter ablations. Six centres (43 %) perform EP procedures on pediatric (< 18 years) population. There was a slight increase in the average amount of EP enabled rooms since 2011 (mean 1.9 up from 1.6). On average, the equipped rooms are used specifically for ablation activity at 75 % capacity in 2015–2016, while in 2011–2012 they were available for ablations only at 70 % capacity (room was at 100 % if used for ablation 5 days/week). Four of the 14 (29 %) centres use the operating room for cardiac rhythm management device implantation, for an average of 3 days per week.

### Ablation procedure types and mapping systems

Diagnostic EP studies and radiofrequency ablations were consistently performed in all participating centres since 2011; ablations using Contact Force catheters have been performed in all centres since 2015. Radiofrequency ablation remains the predominant technique; while the popularity of point-by-point cryoablation and balloon ablation have grown in the last 6 years. Please refer to Table 1 for usage rates of all modalities.

The survey collected data on imaging and electroanatomical mapping systems. CARTO and intracardiac echo were the most widely utilized in 86 % centres, increasing from 78 % of centres in 2011, followed by EnSite NavX-Velocity and EnSite NavX Precision utilized in 71 and 57 % of centres respectively. Only one centre reported the current use of Rhythmia™ and one centre has continued to use LocaLisa since 2011. Two centres in Canada clinically use Robotic Magnetic Navigation systems (Niobe Stereotaxis, St. Louis, MO).

### Personnel

In total, there were 57 full-time physicians (mean: 4.1, range: 0–7) who performed ablations in 2015–2016. 43 % of the responded programs employ 22 part-time physicians, with an average of 1.6 part timers per centre (range 0–12). The number of full-time and part-time EP staff have been increasing (from a mean of 3.5 to 4.1 full-time operators; 1.1 to 1.6 part-time operators per centre in the last 6 years).

There was a trend in more part-time electrophysiologists from 24 to 28 % of total EPs. Ten of the fourteen (71 %) hospitals employ 35 EP fellows, with an average of 2.5 trainees per centre. The ratio of teaching staff (full-time and pro-rated part-time) to trainees is approximately 2:1. We observed a trend of a decreased ratio between staff members and trainees over the polled time.

In 12 of 14 centres, there is an average of 4.6 nurses trained for EP procedures (range: 0–10); and an average of 3 permanent EP (range: 0–7). Seven (50 %) centres employ EP technicians, with an average of 1.5 technicians per centre (range: 0–6). By 2016 non-3D mapping/recording systems became operated predominantly by EP nurses and industry reps, they are the primary operators in 50 and 29 % of the centres respectively, EP techs operated 21 % of the systems in 2016. Table 2 presents complete data on EP labs personnel.

**Table 2:** EP Laboratories Personnel data

	2015–2016			2013–2014		2011–2012		
	Mean	Range		Mean	Range	Mean		Range
Full Time physicians per centre	4.1	(0–7)		4.1	(1–7)	3.5		(0–7)
Part Time physicians per centre	1.6	(0–12)		1.2	(0–5)	1.1		(0–7)
Trainees per centre	2.5	(0–6)		2.9	(0–7)	2.7		(0–5)
Nurses trained specifically for EP	4.6	(0–10)		4.4	(0–10)	n/a		
Permanent EP RN’s	3.0	(0–7)		2.5	(0–6)	n/a		
EP technicians	1.5	(0–6)		1.4	(0–7)	n/a		
Non- 3D mapping systems operated by: N(%)^a^								
EP technicians	3 (21 %)			2.5 (18 %)		9 (50 %)		
EP nurses	7 (50 %)			7 (50 %)		7 (39 %)		
Industry Reps	4 (29 %)			4.5 (32 %)		2 (11 %)		

^a^sums of totals, and percentage of total

### Procedural Data

#### Total procedures

In total, 7106 standard and complex catheter ablation procedures were reported annually for 2015–2016 by all responding centres, an average of 508 ± 270 per centre; this has significantly increased from 2011 to 2012, when the national average obtained by our survey was 421 ± 202 ablations. This is consistent with the increased number of rooms that are solely equipped for ablations, which have increased (mean = 1.9; (1–4) versus mean = 1.6; (1–3)), dedicated to ablations 7 days versus 5 days per week. Centres are steering away from doing implants in the operating rooms towards ablation labs (Table 1).

**Fig. 1:**
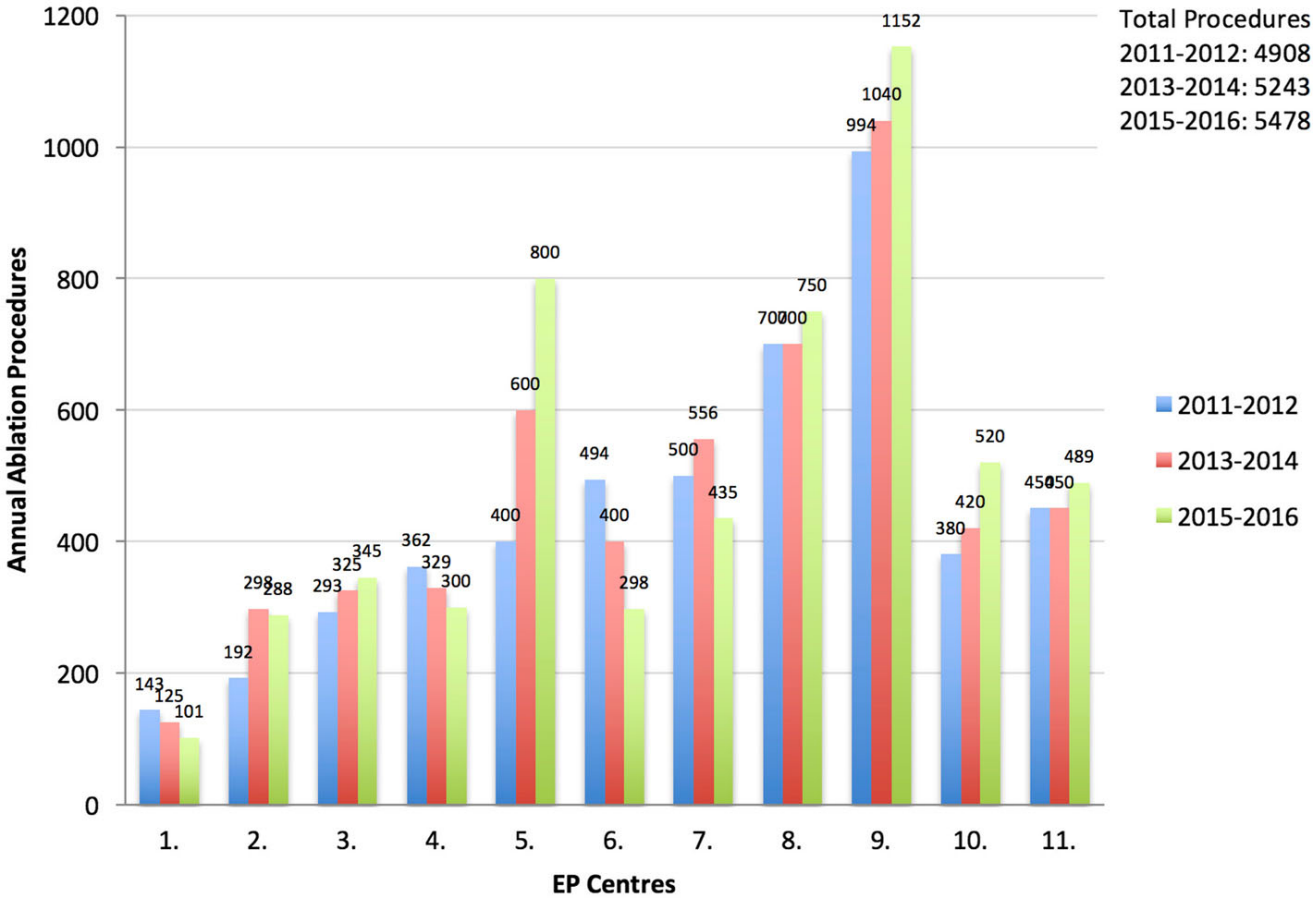
Annual number of ablations per centre in the eleven centres participating in all survey instalments: 2011–2016

If we solely consider the 11 centres that contributed data from 2011, the total number of ablation procedures increased from 4908 in 2011–2012 to 5478 in 2015–2016; with an average of 498 ± 298 versus 446 ± 237 ablations. Figure 1 depicts the trends of the annual number of ablation procedures performed in these eleven centres.

**Fig. 2:**
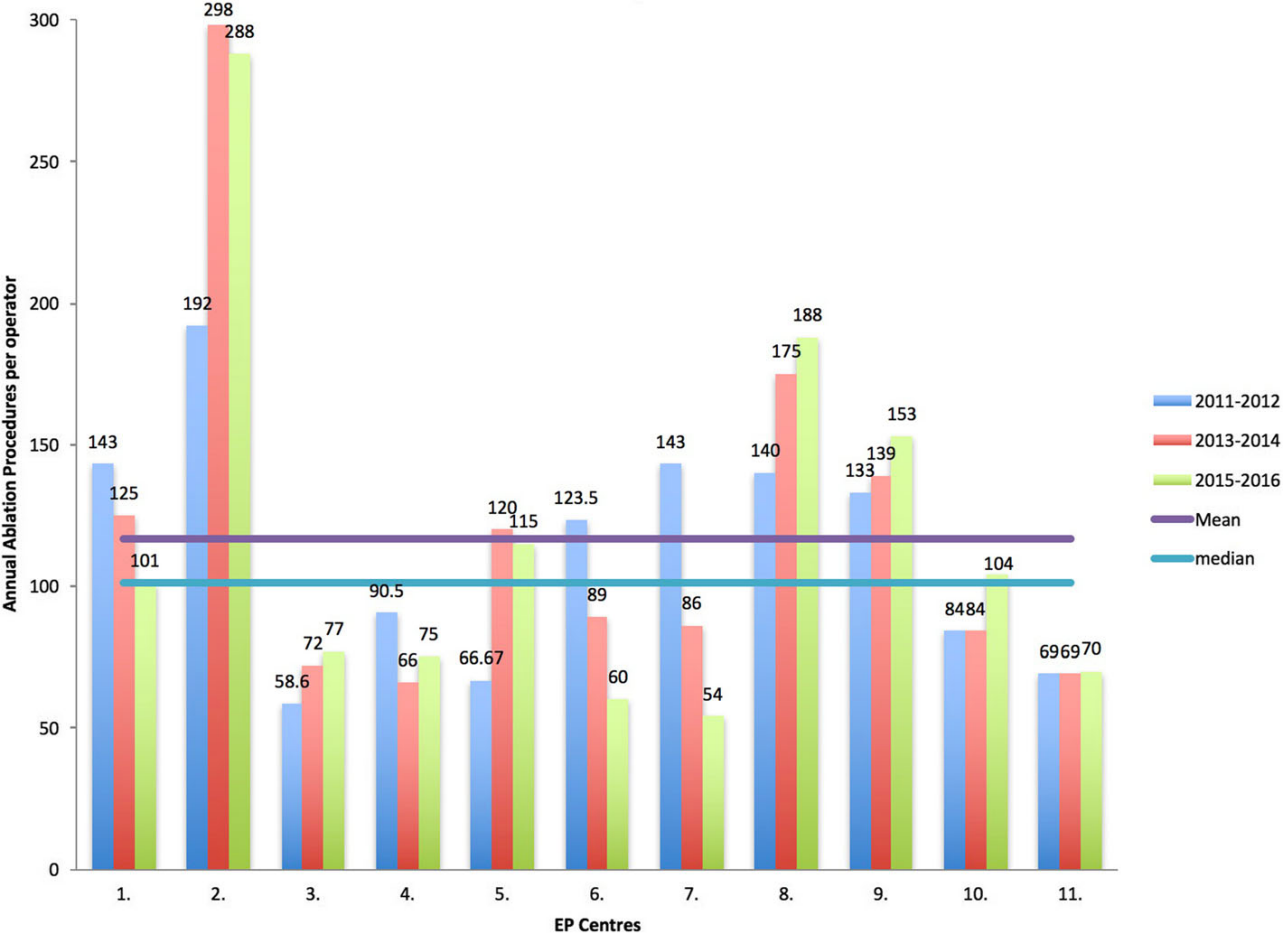
Ablations per operator in the eleven centres participating in all surveys: 2011–2016

On average, each EP specialist was found to perform 117 ± 70 ablations annually in 2015–2016, similar to 113 ± 42 procedures in 2011–2012 (*p* = 0.6); part-time physicians were assumed to perform one half of the ablation volume of full-timers. Figure 2 depicts ablations done per operator.

#### Ablation results by substrates

Substrate-specific data were available from eight centres from 2016, seven centres from 2015; six centres in 2014, five centres in 2013, thirteen centres in 2012, and seven centres in 2011. This comprised a total of 18,864 ablation procedures that were performed over the 6 years (2011–2016), and were included in the following substrate specific analysis. The most frequently performed ablation was targeting AF (PVI) arrhythmia accounting for 36 % of all procedures in 2015-16 (*n* = 2945, mean = 164 ± 85, range = 54–333), 32 % for the years 2013-4 and 30 % in 2011-2. Paroxysmal AF decreased from accounting for the majority (93 %) of all AF ablations in 2012 to 55 % of the cases in 2016, contrary to persistent AF which increased from 29 to 47 %. Chronic success remains consistently > 70 % for paroxysmal AF procedures, and varies between 60 and 90 % for persistent AF.

The second common procedure was the ablation of atrioventricular node reentry tachycardia (AVNRT) accounting for 18 % of all procedures in 2015–2016 (n = 1489), which has decreased from 2011 when this dysrhythmia comprised 23 % of all ablated substrates. The total number of cavotricuspid isthmus (CTI) ablations in 2015-16 was 1473 averaging 98 ± 71 ablation procedures per centre (range 37–327), comprising 17 % of total volume of ablations. Ablation of Atrioventricular Junction and ventricular tachyarrhythmias were the most rarely performed ablations. In 2015–2016 they accounted for 7 and 8 % of ablations respectively, and 8 and 7 % in 2013 and 2014. Table 3 shows the relative frequency of different ablation targets treated by catheter ablation in all centres.

**Table 3:** Volume of Ablation procedures by substrate 2011–2016 reported as percentage of total respondents

	2015–2016	2013–2014	2011–2012
Atrial Fibrillation/Atypical Flutter	36 %	32 %	30 %
AV Nodal Re-entry Tachycardia	18 %	22 %	23 %
Typical flutter	17 %	16 %	21 %
AV Reciprocal Tachycardia	10 %	10 %	11 %
Atrial Tachycardia	3 %	4 %	6 %
Atrioventricular Junction	7 %	8 %	n/a
Ventricular Tachycardia	8 %	7 %	9 %
Total annual ablations in 11 consistently respondent centres	5478	5243	4908

*AV *atrioventricular

#### Procedural complication rates and success rates

Major complications from ablation of accessory pathways (AVRT), AVNRT, and CTI include pericarditis, transient PR prolongation, tamponade, pericardial effusion, stroke, indication for a permanent pacemaker, vascular complications requiring intervention, and death. Minor complications were defined as vascular, hematuria, arteriovenous fistula, pseudoaneurysm, and pain during procedure. The following are self-reported procedural complication and success rates for the 2015–2016 survey. The rate of both major and minor post-procedural complications for the aforementioned substrates were reported as < 10 % in 6 of 7 centres that provided complication rate data, and < 1 % in 1 of the centres. Two centres reported pulmonary embolisms post procedures. The chronic success rate, defined as ‘no recurrence or palpitations were documented other than target arrhythmia’ was greater than 90 % in most (80 %) of reporting centres. Partial success, defined as ‘recurrence with episodes of lesser frequency and severity, requiring the patient to be on the same medications’, occurred with < 10 % frequency in 2 centres, greater than 90 % frequency in 2 centres, and 10–20 % in the remaining centre.

Ablation of AF most commonly leads to minor complications that requires a hospital stay of > 24 h and other complications that don’t require intervention: vascular complications, hematuria, arteriovenous fistula, pseudoaneurysm, and groin pain. Six of 7 centres that reported complications in 2015–2016 had a < 10 % minor complication rate, and one centre reported a < 1 % incidence of minor complications. Major complications of AF: vascular complications requiring intervention more specifically pericarditis, tamponade, pericardial effusion, stroke, need for permanent PM, atrio-esophageal fistula, death, occurred with the same frequency. Chronic success of AF as well as CTI/MAT/FAT ablations were defined as ‘no recurrence or documentation other than target arrhythmia’, it was > 80 % in 2 centres, 71–80 % in 1 centre, > 90 % in 1 centre, and 1 centre reported a 61–70 % rate for 2015–2016. Partial success was < 10 % in 2 centres, 21–30 %, 71–80 %, and > 90 % in 1 centre each.

Ablation of Ventricular Tachycardia had similar major complication rates. Five out of seven centres reported < 10 % minor complication rates, 1 centre reported a < 1 % rate, and in one centre 10–20 % of procedures yielded minor vascular complications of AV fistula and pseudoaneurysm not requiring intervention.

#### Post procedural care

In 75 % of polled centres reported that patients are discharged > 24 h post AF ablation procedure, and in 25 % centres patients are discharged same day. Those undergoing RVOT ablations 50 % were discharged same day, and 50 % >24 h post ablation. In AVRT/AVNRT/CTI ablations 75 % of patients are discharged same day, and 25 % in > 24 h.

#### Ablation of Atrial Fibrillation

Paroxysmal AF was more common than persistent and permanent AF. Paroxysmal AF made up 55 % of annual AF cases in 2016, and made up the majority (93 %) of annual AF cases in 2013. Chronic success, defined as ‘12 months free from documented AF of 30 seconds or more’, was achieved after about 70 % of ablations of paroxysmal AF, ranging from 69 to 71 % between 2013 and 2016. Chronic success was achieved for a range from 60 to 90 % of non-paroxysmal AF ablations. Cases had to be redone between 15 and 22 % of paroxysmal AF, and between 17 and 28 % of non-paroxysmal AF ablations. Table 4 presents the volume of cases and complete technological data as it pertains to PVI procedures.

**Table 4:** Procedural and infrastructure data as it pertains to ablation of Atrial Fibrillation arrhythmia

	2016	2015	2014	2013
Paroxysmal AF	55^a^	58^a^	84^a^	93^a^
Redo cases	22 %	15 %	15 %	22 %
Chronic success	71 %	71 %	69 %	69 %
Non-Paroxysmal AF	47^a^	42^a^	34^a^	29^a^
Redo cases	17 %	22 %	21 %	28 %
Chronic success	60 %	62 %	90 %	85 %
Modality				
Manual	100 %	100 %	100 %	100 %
STXS	17 %	13 %	17 %	17 %
NMARQ (RF)	17 %	13 %	0 %	0 %
Ablation Hardware Availability				
RF irrigated	100 %	100 %	100 %	100 %
RF non-irrigated	33 %	25 %	0 %	0 %
Cryoballoon	100 %	88 %	33 %	33 %
Arctic Front	100 %	75 %	44 %	33 %
Contact Force	100 %	100 %	n/a	n/a
PVAC	17 %	25 %	22 %	33 %

^*^ average annual procedures per year

In 2016, PVI procedures were done under general anesthesia ‘rarely’ in 50 % of the centres, ‘most of the time’ in 33 % of the centres, and used for ‘all’ the cases in 17 % of the centres. Conversely, 50 % use non-general anesthesia most of the time, 38 % use it rarely, and 13 % don’t use it at all. During the years 2014 and 2013, 25 % of the centres used general anesthesia in half of atrial fibrillation ablation cases; 63 % of the centres performed most of the cases under general anesthesia and 13 % of the centres rarely used general anesthesia. Meanwhile, 25 % of the cases used non-general anesthesia in half and most of the cases, and 50 % of the centres only rarely used non-general anesthesia.

Finally, the questionnaire evaluated the type of imaging that was done prior to AF ablation; five centres provided the data for the year 2016. Pre-procedural MRI or CT was done in 76–90 % of the cases by 2 centres, and > 90 % in 3 centres. Pre-procedural TEE was done in > 90 % of the cases in 3 centres, 76–90 % of the cases in 1 centre, and done 1–25 % of the time in the remaining centre. In 2015, 40 % centres conducted pre-procedural CT or MRI imaging on 76–90 % of the cases, 60 % on > 90 % of the AF ablation cases. In both and 2013 and 2014, 75 % centres conducted the pre-procedural CT or MRI imaging, while 13 % did an MRI or CT on 76–90 % of the cases and 13 % of centres do an MRI or CT on less than 25 % of the AF procedures.

## Discussion

This paper presents the trends of types of activities performed and facilities available in Canadian EP practice by reporting results of the 3 administered surveys encompassing data from the years 2011 to 2016. The response rates are comparable with other interventional EP surveys’ response rates including Canada, United States, and Germany [2–4]. Cappato et al. conducted the original web-survey collecting data on practices of catheter ablation of atrial fibrillation, leading to an increase in utilization of this mode of data collection in EP [5]. In our registry, eleven centres have continuously provided their data since 2011 representing 7 of 9 geographic regions where EP services are available in Canada. This, along with improvements in the quality of completed questionnaires, allows this data to accurately represent the country’s current EP landscape.

Overall, the average number of ablation procedures has steadily augmented, from a mean of 421 ± 202 cardiac ablations in 2011–2012 to 508 ± 270 in 2015–2016 per reporting Canadian centre. This growth of procedural volumes is above the population growth in Canada and likely to reflect increased availability of procedural care and increased awareness of the referring community of treatments available. The gains in the amount of procedures were also likely due to improved efficiencies of technology and workflow. The volume of ablations per Canadian centre has surpassed the volumes reported in the latest 3 European registries; in Spain, the mean procedures per centre was 156 ± 126 in 2017 [6], 165.5 □± 127.9 in 2018 [7] and Germany reported a median of 297 ablations per centre [4]. There is a greater number of tertiary and lower volume centres in Europe as opposed to the larger academic centres in Canada, demonstrated by the wide range in the number of annual ablations per centre in Spain (2-568 procedures); and in Germany 11 % of the centres had < 100 ablations per year; and only 60 % of the centres had at least 200 annual catheter ablations. Predictably, we found consistent with the US survey conducted by Hosseini et al., that centres performing > 100 ablations hold the lowest bracket of complications (< 10 %) annually [8].

The growing trends in the number of full and part-time physicians are in line with other reports. The increasing amount of part-time staff in Canada represents community-based cardiologists/electrophysiologists getting access to tertiary based EP labs, and expanding access to community based operators.

The annual ablation volume per operator (117 ± 70 in 2016; 113 ± 42 in 2011) has consistently surpassed the guidelines put forth by the American College of Cardiology, which recommends practicing EP specialists to perform more than 20–50 ablations annually to maintain their competency level [9]. This is consistent with the Canadian Cardiovascular Society guidelines which mandates practicing operators perform 50 ablations annually [10] .

The number of trainees has decreased (2.7 in 2011, to 2.5 in 2016). There is a trend of lesser EP training popularity as EP workforce became more saturated in North America. A training and practice paper by Krahn et al. stated that three fourths of 413 EP fellows who have trained in Canada came from outside the country. Although most foreign trainees return to their country of origin, repatriation is not universal [11].

The survey allows to establish the number of trainees feasible to accommodate per centre. European Heart Rhythm Association requirements state that an electrophysiological training centre should have at least 200 ablation procedures per year; 93 % of the polled centres in Canada performed > 200 ablations per year, whereas 69 % of the German centres reported a > 200 annual ablations volume. A comparison with the American Heart Association, American College of Cardiology, and Heart Rhythm Society advanced training statement revealed that requirements suggested for the United States are similar to those established in Europe [12]. Therefore, for the 2 years training program the average Canadian EP lab may take up to 4 fellows at a time.

The American College of Cardiology, American Heart Association, and Heart Rhythm Society Advanced joint training statement provides a recommendation of 160 catheter ablations as the minimum procedural volume of interventional procedures to achieve and demonstrate competence in clinical cardiac electrophysiology, for the 2-year fellowship training [12]. In reference to these guidelines, when taken the average annual procedures and trainees per centre; it was found that a trainee will finish their competency requirement in almost 1.5 years. The Canadian standard required number of ablation procedures to maintain competency and achieve proficiency are comparable to European standards. The 2017 h/EHRA/ECAS/APHRS/SOLAECE expert consensus statement on catheter and surgical ablation of AF states that outcomes are better at centres that allowed their trainees to perform > 100 procedures. Data report showed improved outcomes for operators with annual procedure volume of at least 25 cases and for centres with an annual procedure volume of at least 50 cases [13].

Pulmonary vein isolation for AF has remained the most frequent ablation target in Canada since 2011, with its absolute rate further increasing. In the year 2016 alone, PVI for AF accounted for 39 % of all annual procedures. This frequency is similar to European registries’ reports of AF being the most targeted substrate, accounting for 47 % of ablations done in Germany in 2015 [2]; 23 % of total ablations done in Spain in 2017 [6] and 26 % of Spain’s total ablations in 2018 [7]. Ablations for ventricular tachycardia is consistently least performed in Canada comprising 8 % of total, comparable to 11 % of ablation targets in Spain, and 10 % of annual procedures in Germany.

The survey has evolved since its original installment to encompass more detailed information on lab infrastructure; we have also started collecting data regarding wait times to see an EP specialist and get a cardiac ablation procedure, see supplement and supplemental Figures 1 and 4. A section solely pertaining to the ablation of AF substrate was introduced in 2013, detailed in the supplement. Furthermore, the survey has been subdivided into 2 parts, the second of which was to be completed if detailed information on types of procedures performed was available. The format of 2 shorter parts of the questionnaire has increased the completeness of each individual part. Furthermore, after the feedback of the first survey’s results publication, success rates and periprocedural complications questions have been introduced. Although this data was still not widely available for many centres it provided important insight into procedural outcomes. The aforementioned modifications to the survey have been implemented in conjunction with the Ablation Committee of Canadian Heart Rhythm Society.

### Limitations

The participation rate does not provide a full representation of all Canadian EP centres; as not all centres responded. The retrospective and self-reported nature of the data may limit analysis. Our aim for future surveys is to include prospective data, and increase participation rate.

## Conclusions

The EP Ablation Registry continues to systematically collect data pertaining to ablation procedures nationwide, the reported data provides a unique opportunity to illustrate trends in Canadian EP practices over last 6 years. The registry demonstrated that the Canadian EP procedural intensity of practice and training is comparable to international standards. This information may provide guidance for making decisions related to staff and trainees capacity for the EP community and for health policy decision makers, as cardiac electrophysiology continues to augment in Canada and worldwide.

## Supplementary Information

**Additional file 1:**

**Additional file 2:**

**Additional file 3:** **Figure S1** Supplement. Waitlist A: wait times to see an electrophysiologist for an initial non-urgent consult.

**Additional file 4:** **Figure S2** Supplement. Waitlist B: wait times between EP consult and date of ablation procedure.

**Additional file 5:**

## Data Availability

all data is available upon request.

## Abbreviations

AF: Atrial Fibrillation
AVNRT: atrioventricular node reentry tachycardia
AVRT: Atrioventricular reentrant tachycardia
CTI: cavotricuspid isthmus
EP: electrophysiology
PVI: pulmonary vein isolation
TEE: Transesophageal echocardiography
