# Supplementary figures and images for: Canadian national electrophysiology ablation registry report 2011–2016
Source: BMC Health Serv Res. 2021 May 6;21:435. doi: 10.1186/s12913-021-06441-0 (PMC8101210; doi:10.1186/s12913-021-06441-0)

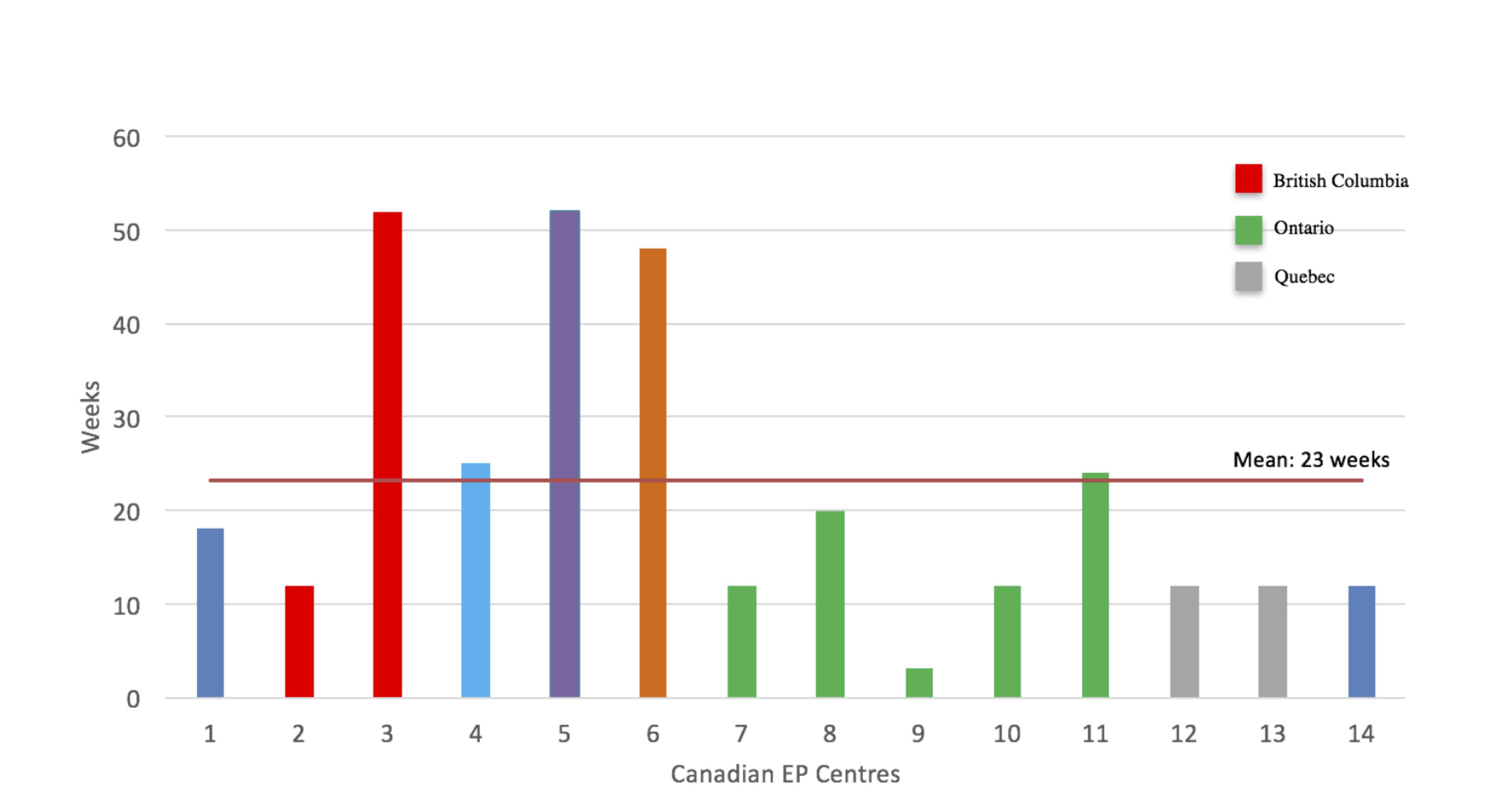

Supplement: Supplementary file 3 — Additional file 3: Figure S1 Supplement. Waitlist A: wait times to see an electrophysiologist for an initial non-urgent consult. [file 12913_2021_6441_MOESM3_ESM.jpg]

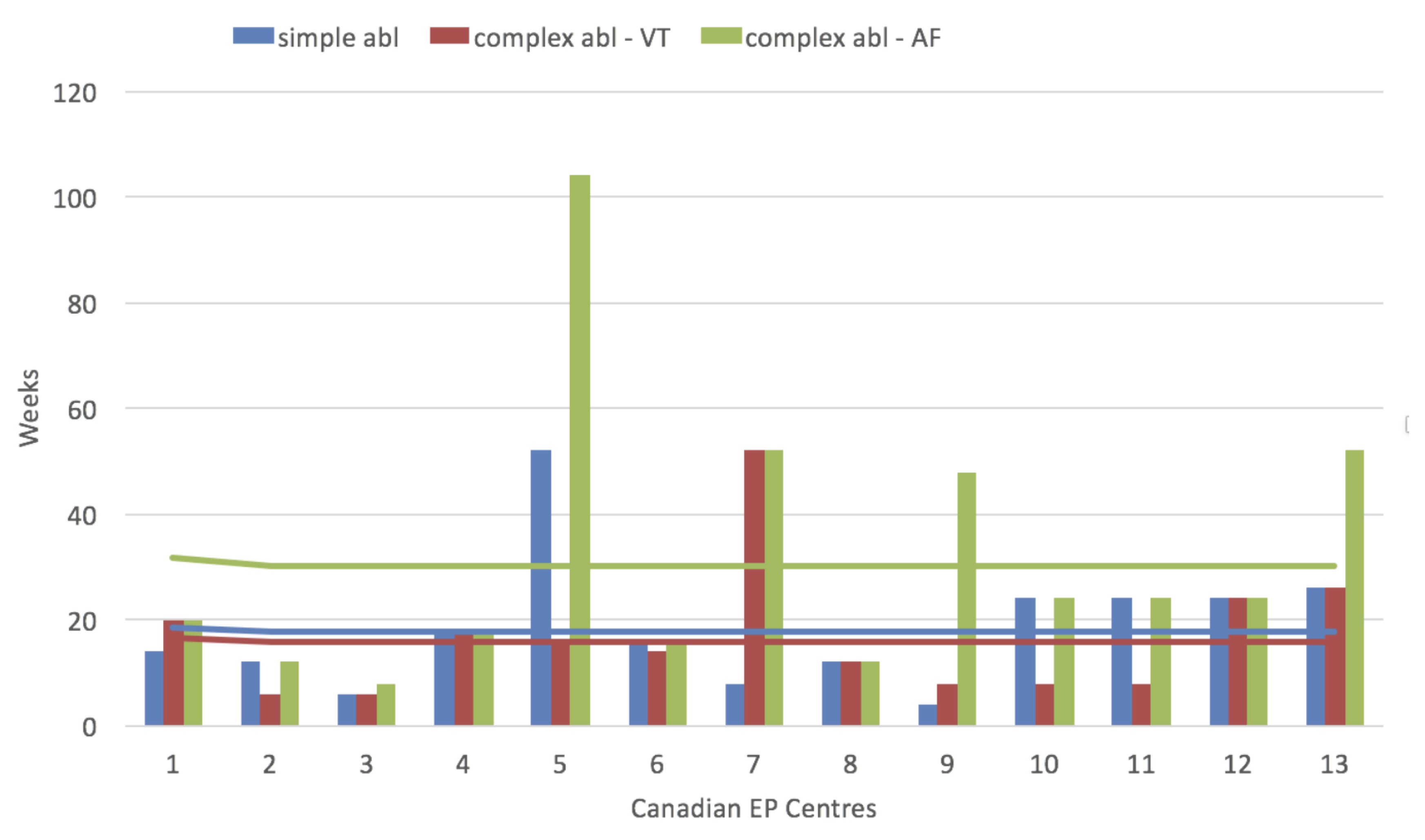

Supplement: Supplementary file 4 — Additional file 4: Figure S2 Supplement. Waitlist B: wait times between EP consult and date of ablation procedure. [file 12913_2021_6441_MOESM4_ESM.jpg]
