# Supplementary material for: Canadian national electrophysiology ablation registry report 2011–2016
Source: BMC Health Serv Res. 2021 May 6;21:435. doi: 10.1186/s12913-021-06441-0 (PMC8101210; doi:10.1186/s12913-021-06441-0)
Supplement: Supplementary file 5 — Additional file 5: [file 12913_2021_6441_MOESM5_ESM.docx]

**Supplement**

**Wait times**

The category of wait times has been introduced to the last survey installment, therefore the subsequent data refers to the years 2015-2016. The mean wait time ‘Waitlist A’ to see an EP specialist for a non-urgent consult from the time of referral was 23 weeks. The two provinces containing centres with the longest wait time to see an EP for an initial non-urgent consult is British Columbia and New Brunswick, where the self-reported wait time is 52 weeks; while the shortest was 3 weeks in Ontario. ‘Waitlist B’ denotes the wait time between a non-urgent EP consult in which a decision for ablation was made. and ablation date (time from the day paperwork was processed by coordinators and ablation date). For a simple ablation procedure, the average wait is 18 weeks (range 4-26 weeks). Complex VT ablation had the shortest average wait time of 16 weeks; ranging 6 weeks in BC and longest time of 52 weeks in Ontario. Predictably, the longest wait time was for a complex ablation of atrial fibrillation arrhythmia, which was 2 years in New Brunswick and the speediest ablation for AF was done 8 weeks after referral in British Columbia. Figures 1 Supplement and Figure 2 Supplement depict all wait times (Waitlist A and Waitlist B respectively).

**Supplemental Figures**

**Figure 1 Supplement.** Waitlist A: wait times to see an electrophysiologist for an initial non-urgent consult.

**Figure 2 Supplement**. Waitlist B: wait times between EP consult and date of ablation procedure.
